# Indo-European loanwords and exchange in Bronze Age Central and East Asia: Six new perspectives on prehistoric exchange in the Eastern Steppe Zone

**DOI:** 10.1017/ehs.2022.16

**Published:** 2022-04-22

**Authors:** Rasmus G. Bjørn

**Affiliations:** Max Planck Institute for the Science of Human History, Kahlaische Strasse 10, 07745 Jena, Germany

**Keywords:** Historical linguistics, archaeology, genetics, Bronze Age, Central Asia, loanwords, Afanasievo, Okunevo, Andronovo, Seima-Turbino, Indo-European, Uralic, Turkic, Old Chinese

## Abstract

Loanword analysis is a unique contribution of historical linguistics to our understanding of prehistoric cultural interfaces. As language reflects the lives of its speakers, the substantiation of loanwords draws on the composite evidence from linguistic as well as auxiliary data from archaeology and genetics through triangulation. The Bronze Age of Central Asia is in principle linguistically mute, but a host of recent independent observations that tie languages, cultures and genetics together in various ways invites a comprehensive reassessment of six highly diagnostic loanwords (‘seven’, ‘name/fame’, ‘sister-in-law’, ‘honey’, ‘metal’ and ‘horse’) that are associated with the Bronze Age. Moreover, they are shared between Indo-European, Uralic, Turkic and sometimes Old Chinese. The successful identification of the interfaces for these loanwords can help settle longstanding debates on languages, migrations and the items themselves. Each item is analysed using the comparative method with reference to the archaeological record to assess the plausibility of a transfer. I argue that the six items can be dated to have entered Central and East Asian languages from immigrant Indo-European languages spoken in the Afanasievo and Andronovo cultures, including a novel source for the ‘horse’ in Old Chinese.

## Graphical abstract

**Figure:**
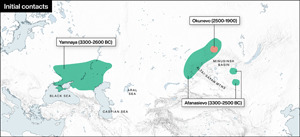

## Introduction

Linguistic contacts between Indo-European and Central and East Asian language families constitute a recurring topic of discussion in historical linguistics, but hardly any consensus on the earliest transfers has been established. Proponents point to similarities and cultural justification (Lubotsky & Starostin, 2003; Napol'skikh, 2001; Helimski, 2001; Pulleyblank, 1966; 1996), while critics note that the proposed transfers cannot be fitted into the known language interfaces (Simon, 2020). Yet can the concrete dating from archaeology and genetics be used to calibrate the relative chronologies of comparative linguistics? And if so, do loanword studies have anything to add to the prehistory of Central Asia?

Since the scientific breakthrough of comparative linguistics roughly 200 years ago, innumerable loanwords have been suggested between Indo-European and Uralic (Carpelan et al., 2001; Simon, 2020; Joki, 1973; Collinder, 1965). Less attention has been given to the possible connections with Turkic (Róna-Tas, 1974; Dybo, 2014; Lubotsky & Starostin, 2003), while contacts with early Chinese civilisation have been a recurring fascination of scholars (Pulleyblank, 1966, 1996; Lubotsky & Starostin, 2003; Blažek & Schwartz, 2017). The lack of consensus is in part due to unsettled chronologies within all language families. It is in this regard of primary importance that Uralic and Turkic have long been considered to belong to a shared linguistic area, commonly known as Ural–Altaic (Janhunen, 2001, 2009; Georg, 2017; Róna-Tas, 1983: 14; Vajda, 2020). In this article, I will present some of the most cited and culturally significant suggested borrowings with updated circumstantial data that may strengthen or reshape previous hypotheses based on lexical evidence alone. This limited set of highly diagnostic correspondences can then be used as a proxy for further dating of contact situations. I will argue that a set of early Indo-European loanwords into Uralic, Turkic and Chinese is of interest not only to historical linguists, but also to anyone wishing to understand Bronze Age interfaces in Central Asia.

### Uralic

**Figure 1.**
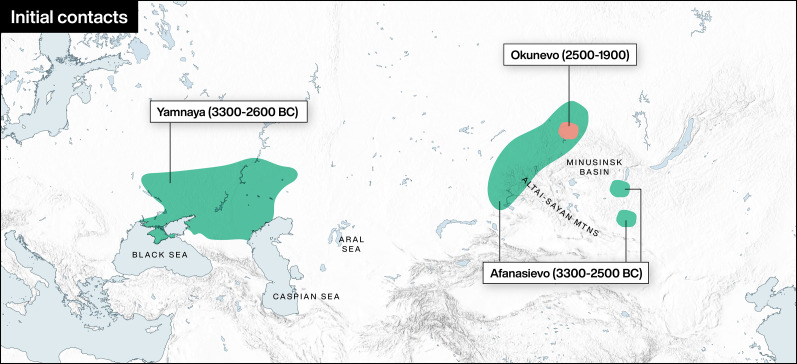
Initial contacts. The earliest Bronze Age cultures in Central Asia.

**Figure 2.**
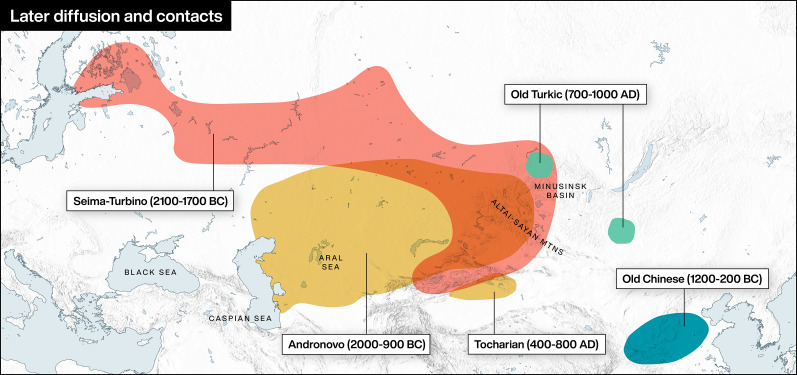
Later diffusion and contacts. Archaeological and linguistic cultural areas in Central Asia mentioned in this article.

The Uralic languages include the national languages Hungarian, Estonian and Finnish, as well as a number of minority languages from Scandinavia to the Yenisei River, including Saami, Mari, Udmurt, Khanty and the Samoyedic languages. The primary branches are, from east to west, Samoyedic, Ob-Ugric, Permic, Volgaic and Balto-Fennic. This geographic distribution may mirror the actual breakup events of the family (Carpelan & Parpola, 2001; pace Salminen, 2001), where a clear east–west divide was quickly established by the ubiquitous Indo-Iranian loanwords in all branches but the more eastern Samoyedic languages (Holopainen, 2019). This division may be further substantiated and contextualised with the demonstration of an early Samoyedic substrate in Tocharian (Peyrot, 2019a; Warries, 2019). The ultimate homeland is still being debated, although scholarly consensus now gravitates towards a relatively recent provenance of the Uralic languages east of the Ural mountains (Grünthal et al., 2022; Häkkinen, 2012: 96–99; Nichols, 2021; Parpola, 2012, 2013; Janhunen, 2009; Aikio, in press-b; Peyrot, 2019; pace Carpelan & Parpola, 2001). A recent estimate puts the dissolution of Proto-Uralic around 2100 BC in association with the contemporaneous 4.2k event and the Seima–Turbino phenomenon (Parpola, 2013: 156–169), a hypothesis that fits the string-like distribution of the western Uralic languages as well as close contacts with the Andronovo (and preceding Sintashta) culture associated with speakers of Indo-Iranic (see Figure 2 and Table 1) (Kuzmina, 2007; Anthony, 2007; Mallory, 1989); Proto-Samoyedic was then either left in or migrated to the area around the Minusinsk Basin. The former origin also fits the association of Proto-Uralic with the Okunevo culture (Janhunen, 2001, 2009; Peyrot, 2019a: 112–114), which came to displace Afanasievo in the Minusinsk basin (see Figure 1 and Table 1) (Mallory & Adams, 1997: 5–6; Mallory, 1989: 223, 263). Native to Central Asia, Okunevo shows cultural traits akin to both Uralic and Altaic (Francfort, 2001) and even genetic links to the pre-Indo-European inhabitants of the Tarim Basin (Peyrot, 2019a: 112; Zhang et al., 2021). I will argue below that the Afanasievo incursion into Central Asia provides a credible explanation for the divergent paths of the Uralic branches and, consequently, a *terminus post quem* for the breakup of Proto-Uralic. Forms for Proto-Uralic follow reconstructions by Aikio (in press-a and in press-b).

### Turkic and wider Transeurasian

The Turkic languages are attested from the first millennium CE, with its earliest and most diagnostic split between Oghuric (going west, extant only Chuvash) and Common Turkic (staying east before spreading across Eurasia, the source of all other modern Turkic languages, including Turkish, Azeri, Uzbek, Yakut, Kazakh and Uyghur) going back to the last centuries BC. Turkic arose in western Mongolia, in close contact with the central steppes (see Figure 2 and Table 1), while Mongolic developed further east (Jeong, et al., 2020; Robbeets et al., 2020: 754–755). The known history of Mongolic is even more recent, but shares with Turkic extensive layers of contact and exchange through millennia in the eastern steppe zone. Together with Tungusic (including Manchu), they form the Altaic branch of the Transeurasian superfamily, originating in the Xinglongwa culture of the West Liao River Basin more than 7000 years ago (Robbeets et al., 2021); this proposed grouping also includes Koreanic and Japonic (Robbeets & Savelyev, 2020). While Proto-Turkic can only be reconstructed internally some 2000 years back, it will be argued that shared loanwords add further weight to the proposition that the proto-language itself may stretch back another 3000 years (Golden, 1998: 16–18), making it contemporary with Proto-Uralic and the Afanasievo incursion.

### Chinese and wider Sino-Tibetan

The modern Chinese languages all descend from Old Chinese that was spoken along the Yellow River (see Figure 2 and Table 1). The language belongs to the wider Sino-Tibetan language family that has been connected with the Yangshao culture (Sagart et al., 2019). Despite early attestations of Old Chinese, the reconstruction of Proto-Sino-Tibetan is still heavily contested (van Driem, 2006). A piece of the puzzle has been sought in loanwords from established linguistic chronologies such as Indo-European. The source language is usually assumed to have been some stage of Tocharian (Blažek & Schwartz, 2017; Lubotsky, 1998; Pulleyblank, 1966; 1996), but other branches have also been considered (Mair, 1990; Beckwith, 2009: 376), and for the present purpose, it is worth paying attention to both the chronology and two main routes of interaction: the northern steppe route and the western desert route (Shelach-Lavi, 2015: 21). Although there is wide consensus that words of Indo-European provenance made their way to early Chinese, the debate is still at an impasse as to exactly when, where and how the loanwords were mediated. I hope to settle parts of the debate with a more plausible source for the introduction of the horse and an earlier and potentially diagnostic borrowing of ‘seven’. Forms for Old Chinese follow reconstructions by Baxter and Sagart (2014).

### Indo-European languages in ancient Central Asia: Tocharian and Indo-Iranic

**Figure 3.**
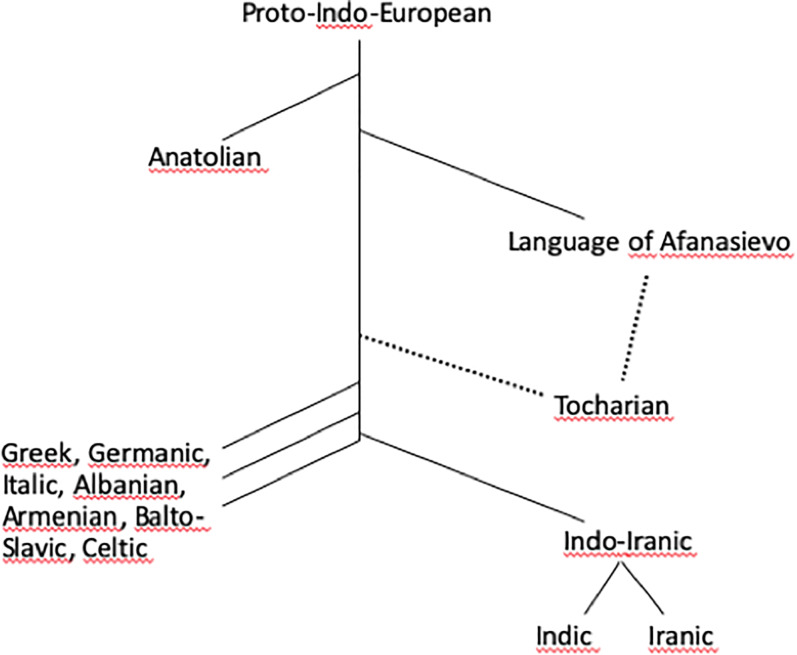
Stylised Indo-European family tree. The branchings on the right are relevant for Central Asian prehistory. It remains to be proven if Tocharian derives directly from the language of Afanasievo

Tocharian is one of only two branches of the Indo-European language family autochthonous to Central Asia. It is attested in the middle of the first millennium AD on the northern rim of the Tarim Basin, southern Xinjiang (see Figure 2 and Table 1). The branch consists of two closely related languages, aptly named Tocharian A and Tocharian B, that through the comparative method can be reconstructed back to Proto-Tocharian, commonly believed to have been spoken in the same region sometime in the first millennium BC (Mallory, 2015: 9; Blažek & Schwartz, 2017). The phonology and extensive agglutinative nominal system of Tocharian have a deviant typology and have played a pivotal role in charting its development (Peyrot, 2019b; Warries, 2019; Bednarczuk, 2015 ; Krause, 1951); a similar change is reflected in the culture that appears as Buddhist, although the conversion can only be few centuries old (Mallory, 2015: 3; Pinault, 1998). It is also widely held to be the second branch to depart from the Indo-European proto-language, after Anatolian (Peyrot, 2019b; Weiss, 2018: 373; Olander, 2018: 185; Anthony & Ringe, 2015: 201, 209; pace Malzahn, 2016). Although the Tocharian migration is commonly associated with the Afanasievo culture (Peyrot, 2019a; Anthony, 2007; Mallory, 2015; Kroonen et al., 2018), the intermediary period, covering more than three millennia, remains difficult to conclusively associate with a particular linguistic tradition. The present paper addresses the earliest part of this issue by identifying unmistakable Indo-European words being transferred from Afanasievo. It should be noted, however, that despite the positive identification of the language of Afanasievo as Indo-European, it does not mean that Tocharian *a priori* belongs to the same branch, and Tocharian could in principle have developed elsewhere from the Proto-Indo-European Yamnaya culture of the Western steppe (see Figure 3).

Indo-Iranic, the second Indo-European branch with a demonstrable prehistoric presence in Central Asia, has been associated with the Sintashta and Andronovo cultures (see Figure 2 and Table 1) (Kuzmina, 2007, 2008). It was probably part of a later sustained and mutually intelligible Indo-European dialect continuum, stretching from the Atlantic into the Steppes until ca. 2100 BC (Koch, 2020: 49–52). After this point the branch further splits into two distinct branches, Indic and Iranic. The earliest attestation of an Indic language is a number of loanwords in the Hurrian language of the Mitanni empire, dated to 1400 BC (Witzel, 2001: 53–55), roughly simultaneously with a continuous oral tradition in Northern India taking form with the Vedic Sanskrit hymns, that only later were committed to writing (Fortson, 2010: 208). The earliest Iranic language, Avestan, has a similar history of early composition but significantly later attestation. Philological and comparative linguistic evidence nonetheless allows a dating of the language to about 1000 BC (Fortson, 2010: 229). The earliest extant piece of writing in the Iranic languages is cuneiform Old Persian from around 500 BC, and the subsequent periods attest to Iranic languages spoken all across Central Asia. The Iranic languages are sorted between Western and Eastern languages, whereof the Iranic languages of the Steppe zone and Central Asian mountains belong to the eastern.

### Indirectly documented languages

Prehistory is an arena with more unknowns than knowns, and the precious pieces of evidence that we have should be used to the fullest extent. What we can say with certainty is that the known linguistic clades have managed or failed to explain the similarities. The linguistic chronologies afforded by the comparative method in the form of language families allow us to evaluate the expansion of the vocabularies in daughter languages, and frequently comparative linguists are left to assume loanwords from an otherwise undocumented language. The minor and rather straightforward assumption that extinct clades were spoken and, indeed, as active in transmitting loanwords and culture as those that survived, essentially dictates that the formulation of such intermediary languages is a worthwhile explanatory framework for otherwise tantalisingly similar forms that may well have been expected to have been borrowed at the relevant time frame, given that they can be substantiated beyond the irregular linguistic correspondences (Andersen, 2003). Because of the inevitable data gaps and asynchronous protolanguages (see Table 1), at the present stage it is necessary to allow a less rigid approach to sound substitutions, while recognising to the fullest extent the need for regular sound laws to ultimately govern historical linguistics. The formulation of potential sound laws and substitutions for hypothesised extinct clades makes them available for testing and revision. This is the reasoning behind the formulation of such indirectly attested linguistic entities as BMAC (Witzel, 2005; Carling, 2005; Lubotsky, 2001; 2020; Palmér, 2019), Temematik (Holzer, 1989; Kortlandt, 2003; pace Matasović, 2014), the European farmer language (Kroonen, 2012; Schrijver, 1997) and extinct dialects of Iranic (Holopainen, 2019; Peyrot, 2018b) and Uralic (Saarikivi, 2006), that are all similarly undocumented languages proposed to account for loanwords not attributable to directly inferable forms. The extinct languages, either within known language families or still unidentified, thus constitute a methodological foundation for historical linguistics.

**Table 1.** Chronological identification of language communities. Rows represent language families, while columns represent time periods. Language community is in bold, archaeological culture in italics, and geographic location underlined. Fields in green are supported by multiple distinct lines of evidence, fields in yellow debated or *a priori* assumed from green fields, while fields in red are unknown (see references in the text).

Before 3500 BC	3000–2000 BC	2000–1000 BC	After 1000 BC
**Proto-Indo-European** Western Steppe	**> Language of Afanasievo** *Afanasievo*	? > **Pre-Proto- Tocharian**	**> Proto-Tocharian** (ca. 500 BC)
	**> Proto-Indo-Iranic** *Sintashta*	**> Proto-Iranic** *Andronovo*	**> Old Iranic**
**Pre-Proto-Uralic**	**> Proto-Uralic** North Central Asia *Okunevo?*	**> Western Uralic** *Seima–Turbino*	**> Western branches**
		**> Pre-Proto- Samoyedic**	**> Proto-Samoyedic**
**Transeurasian** *Xinglongwa*	**> Pre-Proto-Turkic I** Altai	**> Pre-Proto-Turkic II**	**> Proto-Turkic** Altai
**Proto-Sino-Tibetan** *Yangshao*	**> Proto-Sinitic**	**> Old Chinese** (from 1300 BC)	**> Old Chinese**

### The early archaeological cultures: Afanasievo and Okunevo

**Figure 4.**
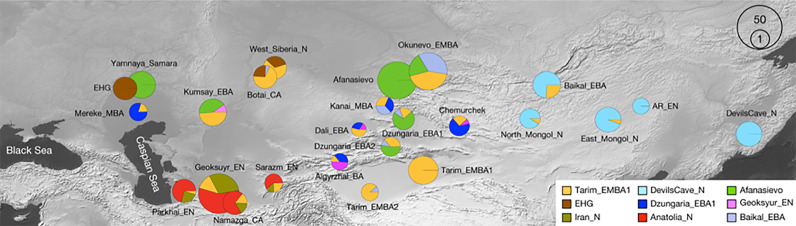
Genetic admixture at the transition from Afanasievo to Okunevo (3300–2500 BC) in Central Asia; note in particular Okunevo_EMBA containing a roughly equal admixture from Afanasievo, a local component, and an eastern migration (map from Zhang et al., 2021).

The Afanasievo culture of the early Bronze Age centred in the Minusinsk Basin and the Altai–Sayan Mountains represents an immigrant community in Central Asia (Honeychurch et al., 2021; Jeong et al., 2020: 891). Although the culture has not left any written artefacts, the continuation of DNA and archaeological culture from Yamnaya of the Western steppes, commonly associated with Proto-Indo-European (Fortson, 2010: 46–48; Anthony, 2007; Haak et al., 2015; Allentoft et al., 2015; Narasimhan et al., 2019; Cunliffe, 2015: 95), has nonetheless compelled researchers to hypothesise that Afanasievo represents the original eastward migration of the Tocharian branch (Anthony, 2007; Kroonen et al., 2018; Peyrot, 2019; Warries, 2019; Mallory, 1989: 263). Despite this widespread optimism, the lines connecting Afanasievo with the Tarim Basin are not straightforward (Mallory, 2015). Afanasievo is succeeded by the Okunevo culture that has been tied to both Uralic and Altaic that also from a linguistic perspective share several traits that indicate they have been in sustained contact in pre-history (see above). Genetically, Okunevo is composed of a mix of Afanasievo, local (Tarim_EMBA1) and an eastern component (DevilsCave_N; figure 4, Zhang et al., 2021), the latter of which I tentatively hypothesise connected to an early Turkic-related ancestry coming out of the West Liao River Basin (Figure 4). Okunevo metallurgical practices are continued in the Seima–Turbino phenomenon (Mei, 2003; Marchenko et al., 2017).

### Structure of the argument

Out of the endless rows of suggested borrowings between Indo-European, on the one hand, and Uralic, Turkic and Chinese, on the other, I hone in on six items that find strong support in the triangulation of archaeological, genetic and/or linguistic discoveries. In the Methods section, I give a short introduction to the comparative method in historical linguistics as well as the theoretical foundation for identifying loanwords. I show how this analysis ultimately relies on the integration of circumstantial evidence through data triangulation as well as the possibility of extinct intermediary languages. In the Data chapter, the items are presented with all the relevant linguistic forms and a discussion of the circumstantial evidence that frames the potential transfer in terms of spatial, temporal and cultural viability. I take the language of Afanasievo as the starting point, but will include the possibility of Tocharian evidence or a later Andronovo and Iranic source when relevant. In the discussion, the stratigraphy of the loanwords, the implications of their identification and possible ways of testing the hypotheses are considered. The novel contribution of the article is the conclusion that, by employing data triangulation of these three independent and recent perspectives, a composite picture emerges that provides dating and location for the otherwise ephemeral pre-proto-linguistic stages of Uralic and Turkic, and to a lesser extent, perhaps also their ancient relations to pre-Proto-Mongolic and Old Chinese.

## Method

Although relying on the testimony of three independent scientific fields, the discussion of data in the presented article departs from historical linguistics. To introduce non-linguists to the governing principles of the discussion, a rudimentary introduction to linguistic comparison is included. The subsequent discussion of the more methodologically challenging task of identifying loanwords is of relevance to linguist readers as well.

### The comparative method

Historical linguistics rely on regular sound laws to establish descent from a shared proto-language (Campbell, 2013; Anttila, 1989). These sound laws are established by applying the comparative method to sets of words in different languages, an example of which can be seen in the cognate set of Table 2. English and Danish regularly agree to an initial *f-* where Latin has *p-*, which, indeed, is one of the characteristics that defines the Germanic branch as distinct from the Italic branch.

**Table 2.** A cognate set between English, Danish and Latin.

English	*foot*	*fish*	*father*
Danish	*fod*	*fisk*	*fader*
Latin	*pēs*	*piscis*	*pater*

When such sound laws can be established to work consistently within a group of languages, they are said to be related. Words in a protolanguage are theoretically deduced and are consequently marked with an asterisk, thus Proto-Indo-European *pods ‘foot’. By collecting all the shared traits in related languages, a more comprehensive proto-language can be reconstructed. The comparative method thus extends the observable developments from attested branches, such as Scandinavian from Old Norse and the Romance languages from Vulgar Latin, into prehistory. The predictability of the method has been confirmed with the discovery of previously hypothesised forms in earlier language stages, including laryngeals in the Anatolian branch of Indo-European and labiovelars in the ancient Greek language of Mycenaean. In prehistory without written artefacts, the comparative method operates exclusively with relative chronologies, where the sound developments can be layered in stratigraphies not unlike an archaeological dig. When a word can be dated with reference to real world items or events it is possible to fit concrete dating to linguistic developments. Only regular sound developments can qualify descent from a common ancestor. Similarities can also be due to contact or chance resemblance and require further analysis.

### Loanwords

All known languages contain loanwords borrowed at different stages of history, just like all types of words may be borrowed (Haspelmath & Tadmor, 2009). Established loanwords can help tie the relative chronologies from different language families and branches together, as the bilateral nature of loanword phenomena provides evidence of both sides of a transfer. Yet since resemblance can arise also through shared inheritance as well as chance, historical linguists commonly stipulate different criteria to determine loanwords (Campbell, 2013: 61–66; Haspelmath & Tadmor, 2009: 43–45; Epps, 2015). These are based on phonological clues (the sound inventories of the language), morphological complexity (the parts of speech usually employed to build words) and cognacy (related words in other languages). Unfortunately, both native speakers and comparative linguists apply the internal logic of the language to nativise foreign elements through a phenomenon known as folk etymology (Anttila, 1989: 92–94): foreign sounds are likened to native sounds and morphology is changed through re-analysis, e.g. English *sparrow grass* for *asparagus*, or *crayfish* from Old French *crevice*. If the internal logic of the folk etymology is accepted and the transfer not directly documented, the possibility of a loanword may erroneously be abandoned. Moreover, these criteria rely on knowledge of a full chronology of the languages involved, and are consequently difficult to apply stringently to comparisons between linguistic isolates, including proto-languages (Haugen, 1950). The comparative method dictates that word comparisons between identified languages follow regular sound correspondences, and the identification of such sound laws is employed to establish loanword directionality. This requires a significant amount of linguistic material to evaluate. At the linguistic proto-stage or with poorly or un-attested languages, however, the number of comparanda (i.e. words that can be compared) decreases to an extent where the regular application of the comparative method becomes untenable (Haig, 2005). As we nonetheless expect borrowings to have occurred, this issue may be remedied by other indicative linguistic phenomena. These include vocabulary events, spreading entire sets of words (e.g. computing, religion, agriculture; Ehret, 2015; Bjørn, 2020) and broader regional distribution of single items entering multiple different languages (e.g. *silk*, *coffee*, *tea*), commonly known as Wanderwörter (Haynie et al., 2014; Epps, 2015: 286). This also ties to a principle of first contact, where novel items are expected to transfer along with their designation at the first encounter, rather than at a later point. The semantic stability of certain items is similarly of importance when comparing languages in prehistory (the items treated here are exceptionally stable, which continuation in Modern English of half of the six Indo-European proto-forms, *seven*, *name* and *mead,* also attests to). Further criteria have been applied that are not solely contingent upon linguistic evidence, and consequently given less weight, including geographical, ecological and cultural clues, that rely on the context of the borrowing (Andersen, 2003). It should be noted that some borrowings are only relevant in this prehistoric setting, and thus find little or no justification from contemporary phenomena. Such is the case with ‘seven’, which finds ample comparative evidence and must be assumed to have been transferred at several independent events (Bjørn, 2020), but in the modern era is borrowed rarely, if ever. This may hold true for other phenomena as well, where the evidence is less obvious. This observation is different from the truism that anything can be borrowed, since the latter carries little evidential weight; rather, it points to the fact that certain things are commonly borrowed, which increases the likelihood of a loanword. There is no historical linguistics without regular sound substitutions, but there are no regular sound substitutions without comparative data, and the dating and likelihood of a particular borrowing is crucial to develop the chronology of both internal and external comparisons in the relevant languages. Although inherently imperfect, the exercise remains to decide if evidence exists to elevate a resemblance beyond chance.

### Data triangulation

Having thus exhausted the possibilities of the inductive comparative method, further analysis relies, as in other historical sciences, on abduction by presenting a hypothesis that accounts for all observations (Ross, 2021; Kiparsky, 2015: 67–68; Anttila, 1989: 25; Watson, 1976). Drawing on evidence from multiple lines of evidence is the bedrock of historical sciences, where the evidence is scarce and typically independent. The identification and formulation of recurring or uniquely complex systems of correlation thus serve as an argument also in comparative historical linguistics. Historical linguistics seldom provide new data to the table, but through the increasingly reliable testimonies of auxiliary sciences new analyses arise from the knowledge of ancient language dynamics. While genetics provides valuable clues about ancient migrations and points of contact, it says little about cultural phenomena. Here, the description by archaeologists will be highlighted when relevant to a particular loanword hypothesis. The present article consequently remains a linguistic analysis that draws on spatial, temporal and narrative interpretations from archaeology and genetics. These three primary axes of prehistoric triangulation, genetic, archaeology and comparative linguistics thus enter a feedback loop, where the evidence evinced from one field can be used to further substantiate hypotheses in the other fields, that all add to the composite picture of prehistory. At the same time, the reliance on different lines of evidence demands that the researcher is prepared to revisit hypotheses based on new and potential contradictory interpretations of data from other fields.

### Methodological protocol

The first step in the current analysis is to identify relevant linguistic data, all of which have been sourced from the vast literature on prehistoric language contacts. In addition to the Indo-European source, the items included here all appear in the earliest recoverable stages of at least two distinct language families, most frequently Uralic and Turkic. As such, they can be classified as Wanderwörter and chance resemblance can be ruled out. The task then is to identify the most likely linguistic stage of transfer, and secondary, to correlate the linguistic stage with the archaeological record. This can be done in two ways, with both the language community and the denoted item. For all items, the linguistic stage is identifiable with the relative chronology of each participating language family and clear *termini ante quem*. Since some of the relevant stages of both Indo-European and Uralic have been associated with archaeological cultures, these can serve as baselines for the dating of the early linguistic stages of Turkic. These are the considerations here committed to ink, but given the nature of the data, they can be nothing more than the initial sketch of the linguistic dynamics resulting from the introduction of the Bronze Age to Central Asia.

## Data and results

### Seven

Proto-Indo-European *(t)septḿ̥ > Proto-Tocharian *ṣäptä > Tocharian A *ṣpät*, Tocharian B *ṣukt*

Proto-Uralic *ćäjć(ć)imä > Proto-Samoyedic *säjʔwǝ, Finnish *seitsemän*

Proto-Turkic *ǯet(t)i > Old Turkic *yeti*, Yakut *sette*, Chuvash *śičĕ*

Old Chinese *ts^h^i̯t > Mandarin *qī*

The entire set has been considered by Napol'skikh (2001: 373), but he fell short of providing a concrete context for the transfers. The argument will be pursued here starting from the original Proto-Indo-European form *septḿ̥. I have previously argued for the possibility that the word was borrowed into Proto-Indo-European as part of a wider numeral spread with the initial *t͡s- as reconstructed for Proto-Semitic (Bjørn, 2020: 61). As the phoneme did not survive in any of the extant Indo-European branches, the onset can reasonably be assumed to have been conflated with initial *s- already in Proto-Indo-European, but it does not preclude the possibility that it actually survived as an allophone into the language of Afanasievo.

The second layer is the reception in Uralic and Turkic. The Uralic form has been the subject of most debate, and a solution with a couple of separate borrowings into the diversifying branches from different Indo-European languages appeared to have reached the level of consensus (Joki, 1973: 313); the Proto-Samoyedic form is thus commonly held to be of Tocharian provenance (Peyrot, 2019a: 100; Janhunen, 1983: 5). This situation has recently been upended by Aikio, who proposes a Proto-Uralic etymology (in press-b: 109–111), although he cannot avoid an Indo-Iranian borrowing into the Ugric branch. Ultimately, Aikio dismisses an Indo-European origin and any resemblance as ‘probably coincidental’, although the question is never properly addressed. Regardless of the ultimate age of the numeral in Uralic, the phonetic objections raised by Aikio are exaggerated (Table 3) as well as contingent upon attested developments within the Tocharian branch. The overall likelihood of undocumented languages provides the objectively difficult, but nonetheless very plausible, scenario of other donor languages. Like most other analyses, Aikio only considers direct contacts between established languages; while prudent, this fails to explore the intricacies and blind spots of proto-linguistic comparisons. The Yukaghir languages, otherwise sharing certain cultural and linguistic traits with Uralic (Aikio, 2014), notably have a decisively different numeral ‘seven’ (Kolyma *pur-ki-* lit. ‘on two’).

The possible Indo-European origin of the Proto-Turkic form, traditionally reconstructed as *yeti, has hardly received mention even in thorough etymological treatments (thus Róna-Tas, 1974: 500; while there is no mention in Blažek, 1999: 106; or Starostin et al., 2003: 959–960). Napol'skikh thus deserves credit for holding on to the comparison, although I have yet to find his substantiation for the transfer. The lack of superficial resemblance makes the form easy to ignore, but the onset, inconsistently reconstructed *y-, *ǯ-, *ǰ-, *dž- to accommodate the various outcomes, most notably Old Turkic *yeti* vis-à-vis Chuvash *śičĕ* and Yakut *sette*, remains one of the major issues in Proto-Turkic phonology (Róna-Tas, 1982: 435; Johanson, 2020: 110–111). Attempts to provide an internal etymology or connect the form with the other Altaic/Transeurasian languages are semantically and phonologically strained and, at best, less obvious than a borrowing (Blažek, 1999: 106). The rest of the Turkic form, *-*et(t)i* is simpler than in Uralic, but this may tentatively be ascribed to at least another millennium of pre-proto-linguistic development before Proto-Turkic emerges in the last centuries BC (see below). Proto-Turkic *ǯet(t)i thus conceivably belongs with Uralic.

The last form proposed by Napol'skikh is Old Chinese *ts^h^i̯t that in phonological principle resembles the Uralic and Turkic forms and consequently could be considered as part of the same phenomenon. Unfortunately, the circumstantial evidence is less obvious as both the spatial and temporal vectors remain unsettled in the wider context of Sino-Tibetan, where a common root has been suggested (Matisoff, 1997: 10–11, 84–90; Schuessler, 2007: 419), but with persistent difficulties. Ultimately, the possibility of a borrowing of Indo-European provenance may be strengthened by further borrowings belonging to the same contact phenomenon as Uralic and Turkic.

All proposed loanwords thus share a similar phonological structure (see Table 3) with an affricated onset, a front vowel and a medial or final dental. The presumed closest contacts Uralic and Turkic share further syllables, that for Uralic also contain a labial element, that may be compared with the vocalised bilabial nasal in Proto-Indo-European. The typologically most difficult transfer is the affrication of a borrowed *s-. Such a phenomenon can nonetheless be observed in the German Rhein dialect of Cologne, where loanwords with initial voiceless *s-* regularly become affricated to *ts-*, e.g. *t͡soldat* ‘soldier’ (Heike, 1964: 45, 85–86, 132). As all three potential borrowing languages agree to the affricate, it was probably present already at the initial transfer. I submit three different solutions to this problem, while noting that it may have arisen through a combination:

1. a retention of the hypothetical Proto-Indo-European onset *t͡s-;
2. a special development in an extinct branch of the language of Afanasievo; or, alternatively,
3. the reception of an unfamiliar form in the pre-proto stage of either Uralic or Turkic.

**Table 3.** Sound correspondences of possible loanwords for ‘seven’.

Proto-Indo-European	*(t)s	*e*	*pt*	*ḿ̥*	
Proto-Uralic	*ć	*äj*	*ć(ć)*	*i*	*mä*
Proto-Turkic	*ǯ	*e*	*t(t)*	*i*	*-*
Old Chinese	*tsh	*i̯*	*t*	*-*	*-*

I provide the following tentative sound developments (underlined) to account for the attested forms (→ means ‘was borrowed into’), noting both Turkic and Chinese may have been adopted from PPU 1 or PPT 1, respectively:

Proto-Indo-European *(t)septḿ̥ > language of Afanasievo *(t)sjeptem

→ Pre-Proto-Uralic (PPU) 1 *(*t)sjeptim* > PPU 2 *ćäjt^j^t^j^im-ä > Proto-Uralic *ćäjć(ć)imä

→ Pre-Proto-Turkic (PPT) 1 *(*d)zjeptim >* PPT 2 *ǯettim > Proto-Turkic *ǯet(t)i

→ Pre-Proto-Chinese (PPC) 1 *(*t)sjeptim >* PPC 2 *ts^h^itti > Old Chinese *ts'iĕt

### Name and fame

Proto-Indo-European *h_3_neh_3_mn-k̂leu̯os ‚fame‘

> Proto-Tocharian *ñæm-klyäwæ ‚id.‘ > Tocharian A *ñom-klyu* ‚id.‘, Tocharian B *ñem-kälywe* ‚id.‘

Proto-Turkic *(at) kü ‘fame, reputation’ > Old Turkic *kü* ‘id.’, Uighur *(at) kü* ‘renown, glory’

Proto-Uralic *nimi ‘name’

Proto-Yukaghir *nime ’name’

Chukchi *ninn* ‘name’

Old Japanese *na-* ‘person, name’

Ainu *namup* ‘name’

The concept of ‘fame’ was evidently a persistently important phenomenon in Proto-Indo-European (as witnessed by cognates in Tocharian, Greek and Vedic; Watkins, 1995: 65, Adams 2013: 288), and thus probably also in the language of Afanasievo. The word is a compound of two common nouns of some significance in Proto-Indo-European: the first member is the root also of English *name*, while the second member became the endonym of the *Slavs*.

Pinault proposes that the entire concept was adopted from “Tocharian (A)” into “Ancient Turkic” (1998: 358–360; Carling, 2005: 55) based on the phrase in Uighur with the first member substituted by the native Turkic word *at* ‘name’ (see Table 5). It is nonetheless unclear exactly what stage Pinault envisions (I have not been able to ascertain a cognate in Chuvash or the Oghur branch more broadly), but the word is attested frequently already in Old Turkic, where its usage appears to be waning (Clauson, 1972: 686). The adoption of Buddhism may, with Pinault, hesitantly be taken as a *terminus ante quem* for a borrowing from the Tocharian culture, although the word evidently was in use during that period as well. The phonology of Proto-Turkic prohibits initial consonant clusters, and a transfer already from Proto-Tocharian seems just as plausible; even earlier dates remain a hypothetical possibility, contingent upon the development of *k̂leu̯os ‘fame’ in Proto-Tocharian or the language of Afanasievo ca. 3000 BC, and the reception and development into Proto-Turkic *kü ‘fame, glory’ only securely datable to the first millennium AD.

Inversely, only the first member is present in Proto-Uralic, where *nimi ‘name’ has been one of the primary bones of lexical contention for the Indo-Uralic hypothesis (Helimski, 2001; Kloekhorst & Pronk, 2019). Most problematic has been the *i*-vocalism, which may prove to be insurmountable for the direct comparison with Tocharian, although the specific choice of *e*-grade in Tocharian could be an important clue to an early innovation also in the language of Afanasievo (Meier & Peyrot, 2017: 18–19, treating the word for ‘honey’ in Tocharian and Chinese, make the case for an intermediary and old stage of a fronting of *e in certain conditions). A development in an extinct branch of the language of Afanasievo, and evidently not found in Tocharian, could salvage both words (see Table 4). Close to Uralic is Yukaghir, while further afield lies the Palaeo-Siberian language Chukchi (still with *i-*vocalism). An Indo-Iranic source for either of the forms above can be excluded, as neither a-vocalism nor palatalisation is visible, cf. the Vedic (reversed) form *śrútya-nā́man*.

The situation with the Japanese and Ainu (a language isolate now spoken in northern Japan) comparanda is less clear-cut (see Table 6). Here, the *a*-vocalism suggests that a potential transfer is to be found in connection with the later Andronovo expansion, but the simple structure of the words renders chance similarity a possible explanation, contingent upon potential internal derivation in Japonic (Robbeets, 2005: 175). ‘Person’ can be viewed as semantically contiguous with ‘name’ as a marker of social status, and the less contentious lexical items reaching early Japonic (see ‘honey’ and ‘horse’ below) make it difficult to exclude connections with the Central Asian exchange system. Demonstrable chance status thus remains to be substantiated (pace Simon, 2020: 248-49).

**Table 4.** Sound correspondences of possible loanwords for ‘name’ with i-vocalism.

Proto-Indo-European	* h^3^n	*eh^3^*	*m*	*n*	*kl̂*	*eu̯os*
Proto-Uralic	*n	*i*	*m*	*i*	—	—
Proto-Yukaghir	*n	*i*	*m*	*e*	*—*	*—*
Chukchi	*n*	*i*	*nn*	*-*	*—*	*—*

Proto-Indo-European *h_3_neh_3_mn-k̂leu̯os > language of Afanasievo *ni(:)min-k.

→ Proto-Uralic *nimi

→ Proto-Yukaghir *nime

→ Chukchi *ninn*

**Table 5.** Sound correspondences of the possible loanword for ‘fame’ in Old Turkic (Pinault, 1998).

Tocharian A	*ñom*	*kl*	*yu*
Old Turkic	*(at)*	*k*	*ü*

**Table 6.** Sound correspondences of possible loanwords for ‘name’ with a-vocalism.

Proto-Iranic	*n	*ā*	*m*	*an*
Ainu	*n*	*a*	*m*	*up*
Old Japanese	*n*	*a*	*—*	—

### Sister-in-law

- Proto-Indo-European *ĝlh̥_3_-(wos) ‘sister-in-law’
  > Latin *glōs*; Old Church Slavonic *zŭlŭva*; Ancient Greek *γάλως*; Phrygian (according to Hesychius) *γɛλλαρος*; Armenian *tal* (no attested Central Asian forms)
- Proto-Uralic *käliw ‘brother- or sister-in-law’
- Proto-Yukaghir *kel'il ‘brother-in-law’
- Proto-Turkic *kälin ‘bride, sister-in-law’
- Proto-Tungusic *keli ‘brother-in-law’

Unlike all other items treated in this article, a Proto-Indo-European provenance is far from given as the cognate set appears regionally confined to Europe; a connection with Sanskrit *giri* was commonly adduced (Mayrhofer, 1986–2001: 487–488), but the initial *g* does not show palatalisation as expected from a cognate and the philological assessment allows for different interpretations (Griffith & Lubotsky, 2009: 118–121). The word is thus not securely attested in an Indo-European language of Central Asia, even though an Indo-European source is often assumed for the word in Uralic (Koivulehto, 1994: 140).

The set of comparanda for a marital relation (see Table 7) is a recurring feature in etymological treatments for the Central and East Asian languages (Räsänen, 1969; Redéi, 1988–1991; Nikolaeva, 2006), and parts of the spread seem fairly settled: Proto-Yukaghir borrowed the word from the Proto-Samoyedic branch of Uralic (Aikio, 2014: 70), on account of the semantic development to ‘brother-in-law’, which then may be further connected with Tungusic.

**Table 7.** Sound correspondences of possible loanwords for ‘sister-in-law’.

Proto-Indo-European	* ĝ	*-*	*l*	*h̥^3^*	*(wos)*	‘sister-in-law’
Proto-Uralic	*k	*ä*	*l*	*i*	*w*	‘sister-in-law’ /‘brother-in-law’
Proto-Yukaghir	*k	*e*	*l’*	*i*	*l*	‘brother-in-law’
Proto-Turkic	*k	*ä*	*l*	*i*	*n*	‘sister-in-law’
Proto-Tungusic	*k	*e*	*l*	*i*	*—*	‘brother-in-law’

Doerfer rejects external relations for the Turkic word in favour of an internal formation to the verbal root *käl- ‘to come’ (Doerfer, 1963–1972: 666–667). Notwithstanding the obvious risk of folk etymological association, this analysis could in theory be used to pinpoint Proto-Turkic as the ultimate source of the word, although it is difficult to see how the word then could have spread west sufficiently early with the dearth of Iranic comparanda. Additional comparanda in Proto-Semitic *kallatu ‘daughter-in-law, bride’ (where *-*at-* is the Semitic female ending) and perhaps North Dravidian (*qali ‘female relative’) give further weight to a (south)western origin (Illič-Svityč, 1971: 295–296; Bjørn, 2017: 55–56).

Proto-Indo-European *ĝlh̥_3_(wos) > language of Afanasievo *keliwos

→ Proto-Uralic *käliw

→ Proto-Yukaghir *kel'il

→ Proto-Tungusic *keli

→ Proto-Turkic *kälin

Proto-Uralic and Proto-Turkic appear closely related, and may conceivably have been in close contacts at the initial adoption of the western relationship term.

### Honey

Proto-Indo-European *medhu- ‘honey, sweet’

> Proto-Iranic *madu- > Avestan *maδu*

> Proto-Tocharian *ḿätä > Tocharian B *mīt*

Proto-Uralic *meti

Old Chinese *mit

Old Japanese *mitsu*

Proto-Turkic *bal > Old Turkic *bal*, Chuvash *pïl*

Proto-Mongolic *bal

In order to make sense of the various Central and East Asian forms, we again will have to consider all Indo-European descendants in Central Asia. It should be noted that, although the word is not attested in Proto-Samoyedic, it is widely believed to have been secondarily lost, prompting scholars to assume that it was known already in Proto-Uralic (Joki, 1973: 283–285; Carpelan & Parpola, 2001: 119). The Chinese form is a relatively late entry, only attested linguistically as well as in the archaeological records in the latter part of the first millennium BC (Meier & Peyrot, 2017). While an Indo-Iranic source for Uralic or Chinese can be excluded owing to the vocalism in the recipient languages, the oft-cited Tocharian origin (Lubotsky, 1998: 379; Schuessler, 2007: 383) is unlikely to have developed the *i*-vocalism sufficiently early, and a proper explanation is still wanting (Jacques, 2014; Meier & Peyrot, 2017). It is commonly agreed, however, that both Uralic and Chinese (and Japanese) ultimately derive their ‘honey’ words from a descendant of Proto-Indo-European *med^h^u- (see Table 8). Curiously, the western Uralic word for ‘bee’ is a clear borrowing from early Proto-Indo-Iranic (Holopainen, 2019: 139–142) and it is tempting to assign ‘honey’ to the same stratum, but the lack of diagnostic sound changes makes other early Indo-European sources possible too. Carpelan and Parpola here provide a comprehensive archaeolinguistic analysis, drawing also on the possible adoption of ‘pot’ and ‘water’ from the Indo-European source to assign the borrowing to Proto-Uralic (2001: 122), but their spatial and temporal vectors are tied to the Volga already at 5000 BC, which is incompatible with the Seima–Turbino phenomenon now commonly associated with the spread of western Uralic (see above).

Proto-Turkic *bal cannot have been borrowed from the same source, and the word probably only entered Turkic with the Iranic Andronovo expansion (Mayrhofer, 1963: 571), ultimately with the same word *med^h^u- as in Uralic and Chinese, although at a later point still early enough to predate the Turkic development of *m- > *b (see Table 9). This requires the intervocalic dental (*d^(h)^) in the source language, probably the hypothesised Old Steppe Iranian, to have approached a lateral pronunciation (*l).

**Table 8.** Sound correspondences of possible loanwords for ‘honey, sweet’ with i- and e-vocalism.

Proto-Indo-European	* m	*e*	*d^h^*	*u*
Proto-Uralic	*m	*e*	*t*	*i*
Old Chinese	*m	*i*	*t*	*-*
Old Japanese	*m	*i*	*ts*	*u*

Proto-Indo-European *med^h^u- > language of Afanasievo *medu-

→ Proto-Uralic *mete

→ Old Chinese *mit

→ Old Japanese *mitsu*

**Table 9.** Sound correspondences of possible loanwords for ‘honey, sweet’ with a-vocalism.

Common Iranic	* m	*a*	*δ*	*u*
Proto-Turkic	*b	*a*	*l*	*-*
Proto-Mongolic	*b	*a*	*l*	*-*

### Gold, bronze, and copper

Proto-Indo-European *h_2_ues-h_2_ ‘gold’

> Proto-Tocharian *ẃäsa ‘gold’ > Tocharian A *wäs* ‘gold’, Tocharian B *yasa* ‘gold’

Proto-Turkic *yez ‘copper (alloy); bronze’

Proto-Samoyedic *wesä ‘iron’

Finnish *vaski* ‘bronze, copper’

Internal irregularities in the Uralic group of words denoting a metal, either ‘iron’ (e.g. Hungarian *vas* and Proto-Samoyedic *wesä) or ‘bronze/copper’ (e.g. Finnish *vaski*) point to an early Wanderwort phenomenon rapidly spreading in the diversifying dialects (Aikio, 2015: 43). This perfectly fits the Seima–Turbino phenomenon, that saw groups of hunter–fishers in the forest zone west of the Altai mountains adopt a bronze industry and rapidly spread it laterally across the entire Eurasian forest and forest-steppe zone, including western China (Grünthal et al., 2022: 11; Cunliffe, 2015: 142–143; Mei, 2003; Goody, 2012: 167). Although the source of the word in Central Asia has been the centre of some debate, the combined evidence clearly points to a borrowing from a continuation of Proto-Indo-European *h_2_ues-h_2_ ‘gold’, originally derived from the meaning ‘shining’, which is continued in Proto-Tocharian ‘gold’. The semantic shift from ‘gold’ to ‘copper, bronze’ (and later ‘iron’) does need justification, but may conceivably have been transferred as ‘metal’ more broadly since Afanasievo introduced the novel concept of metallurgy (see Table 10). The Turkic form is commonly believed to derive from early Tocharian B *yes ‘gold’ (Rybatzki, 1994: 223–225; Jankowski, 2013: 533; Dybo, 2007: 125; Róna-Tas, 1974: 504); note also further borrowings into Mongolic (*ces* ‘copper’) and a western Uralic language (Mordvin *śerä* ‘copper’; Clauson, 1972: 982). Association of the initial *y- in Turkic with the outcome in Tocharian B seems straightforward, but it does not preclude the viability of an earlier transfer into Proto-Turkic, that notably lacks the labial glide *w*. This earlier interpretation finds some substantiation in the otherwise curious semantic transfer of Tocharian B ‘gold’ to Old Turkic ‘brass’ well into the Iron Age, since the borrowing must have happened sometime between Proto-Tocharian (ca. 500 BC) and the attestation of Tocharian B in the middle of the first millennium AD, when Proto-Turkic already had words for gold (*altun) and copper (*baqir).

**Table 10.** Sound correspondences of possible loanwords for ‘copper, bronze, metal’.

Proto-Indo-European	* h_2_u	*e*	*s*	*h_2_*	‘gold’
Finnish	*v*	*a*	*sk*	*i*	‘copper’
Proto-Samoyedic	*w	*e*	*s*	*ä*	‘iron, metal’
Proto-Turkic	*j	*e*	*z*	*—*	‘copper, brass’

Proto-Indo-European *h_2_ues-h_2_* ‘gold’ > language of Afanasievo *ẃesa ‘gold’

→ Proto-Turkic *yez ‘copper (alloy); bronze’

→ Proto-Samoyedic *wesä ‘iron’

→ Finnish *vaski* ‘bronze, copper’

### Horse

Common Iranic *bāraka- ‘beast of burden, horse’

> Ossetic *bajrag* ‘foal’, Yazghulami *varag* ‘horse’, Shughni *vōrǰ* ‘horse’

Old Chinese *mˤraʔ ‘horse’ > Mandarin *mǎ*

Proto-Mongolic *morin ‘horse’

Old Chinese *mˤraʔ (and further Mongolic *morin* as well as additional borrowings into Korean, Japanese, and Tungusic) has been connected with Celto-Germanic *márkos ‘horse’ (Bradley, 2016: 8; Pulleyblank, 1996: 14; Mallory & Adams, 1997: 274). While the phonological similarity may be tantalising, the spatial chasm between Central Europe and East Asia can only be amended by the assumption that the word was carried into Central Asia by Afanasievo, Tocharian or Indo-Iranian. Unfortunately, there is little evidence that the word is of Proto-Indo-European date, as Proto-Celtic *markos is thought to be a borrowing from an unidentified source and only secondarily spread to Proto-Germanic (Matasović, 2009: 257; Koch, 2020: 105); at best, the forms may be related, but necessarily through intermediaries. I tentatively suggest a more plausible source that demonstrably has been spoken in Central Asia: Proto-Iranic *bāra-ka- ‘beast of burden, horse’, internally derived from the meaning ‘carry’ with continuants in Ossetic *bajrag* ‘foal’ (a descendant of the Iranic steppe languages, spoken in the Caucasus), Yazghulami *varag* ‘horse’ and Shughni *vōrǰ* ‘horse’ (both spoken in the Pamir close to the Ferghana valley; Witczak & Novák, 2016: 57; Morgenstierne, 1974: 85–86; Abaev, 1958: 232). The pivotal nasalisation of the initial in Iranic (*b- → *m-) can be motivated in the target language where Schuessler remarks a tendency to receive foreign initial *b- as Old Chinese *m- in front of *r* or *l* (2007: 66–67; cf. Matisoff, 2003: 133), no doubt abetted by the already spirantised pronunciation of *b- in early Iranic (Steblin-Kaminskij, 1999, 33). An Iranic source thus satisfies spatial, temporal, and linguistic vectors for a loanword into Old Chinese (see Table 11).

**Table 11.** Sound correspondences of possible loanwords for ‘horse’.

Common Iranic	*b	*ā*	*r*	*a*	*k*	*a*
Old Chinese	*m	ˤ	*r*	*a*	*ʔ*	*—*
Proto-Mongolic	*m	*o*	*r*	*—*	*—*	*in*

Common Iranic *bāraka- ‘beast of burden, horse’

→ Old Chinese *mˤraʔ ‘horse’ > Mandarin *mǎ*

→ Proto-Mongolic *morin ‘horse’

## Discussion

For the items treated in this article, the mass of circumstantial evidence demands that the linguistic evidence, imperfect though it may be, is recognised as a unique contribution to our knowledge of prehistoric contact phenomena. While most proposed prehistoric loanwords are bilateral and consequently difficult to substantiate beyond reasonable doubt, the six items presented here can all be (a) tied to a particular Bronze Age phenomenon, (b) found in multiple different contiguous language families and (c) phonologically explained with a minimal number of assumptions. Although several details still need further investigation, and indeed, it may never be possible to pinpoint the exact time of place of borrowing for each item (see Table 12), they nonetheless give voice to a period of great societal transformation in Central and East Asia.

**Table 12.** Relative chronology of Bronze Age borrowings into Uralic, Turkic, and Old Chinese.

Dating	Criteria	Uralic	Turkic	Old Chinese
Early (3300–2500 BC?)	Proto-languages, not from Iranic	*seven, name, sister-in-law (honey?)*	*seven, sister-in-law, metal (fame?)*	*seven*
Middle (2500–1000 BC?)	Iranic source/irregular correspondences	*metal (honey?)*	*honey, (fame?)*	*horse*
Late (1000– 0 BC)	attested introduction	*—*	*(fame?)*	*honey*

Triangulation allows us to assume an Indo-European speech community in Afanasievo. Genetics and archaeology provide strong continuation of Yamnaya ancestry and culture both in Afanasievo and the later Andronovo horizon, that conclusively has been associated with the spread of the Iranic languages. Comparative linguistics now adds to this picture by pointing to a robust set of early borrowings of Bronze Age items of Indo-European origins into native Central and East Asian language families. With the one assumption that the Afanasievo culture consisted of speakers of an early Indo-European language, it is possible to reevaluate proposed loanwords in the region, in particular with Turkic and Uralic. The language of Afanasievo is unlikely to have remained a monolithic speech community for very long, stretching all the way to the southern Khangai Mountains (central Mongolia), about 1500 km beyond the Altais (Jeong et al., 2020: 892; Honeychurch et al., 2021), and dialectal variation provides a welcome solution to the recalcitrant problems that still haunt the otherwise tantalising comparanda.

### Seven

Although some details remain to be sorted out, all available lines of evidence point to the continuation of the general spread of a numeral ‘seven’ into Central Asia. As such, the Uralic (and not Yukaghir) and Turkic (and not Transeurasian) forms support each other in contrast to their respective linguistic spheres and, including Chinese, with shared developments from the Indo-European source form. I consequently suggest that this borrowing is an Uralo-Turkic phenomenon, transferred at an early stage of contact with the language of Afanasievo; this word was possibly borrowed further into Old Chinese, perhaps through the language of the local cosmopolitan people of the Tarim Basin (Zhang et al., 2021). As an exclusively linguistic contribution, early Neolithic and Bronze Age exchanges across Eurasia saw a rapid spread of a formalised decimal system, mostly grammaticalising former idiosyncratic counting practices, but also borrowing foreign numerals, in particular ‘seven’ (Calude, 2021; Mallory & Adams, 1997: 398; Helimski, 2001: 190–192; Janhunen 2012; Bjørn, 2020, 2017: 141). It is very likely that the precipitation of the numeral systems across Eurasia is tied to increased exchange, no doubt to a large extent with the new precious metals (Mei, 2003), ultimately an extension of the Balkan–Carpatho metallurgical network, reaching the Volga in the fifth millennium BC (Cunliffe, 2008: 154–156; Goody, 2012: 167–168; Rehren et al., 2021: 7). Reconstruction to Proto-Uralic and Proto-Turkic indicates that ‘seven’ belongs to the earliest stratum of loanwords of Indo-European provenance in Central Asia, most likely propagated by the Afanasievo incursion.

### Name-fame

The prestige driven hierarchical society introduced from the west (Honeychurch et al., 2021: 13) is an apt vehicle for the concept of glory and personal distinction, quite literally with the prospect of ‘making a name for oneself’ in the context of social reorganisation. Unlike with ‘seven’ above, the borrowings in Turkic and Uralic may be independent, but nonetheless attest to a widespread phenomenon taking on Wanderwort status. This transfer may find further substantiation in relation to the simultaneous borrowing of ‘sister-in-law’ in a period where social position and marriage alliances become increasingly institutionalised. Since ‘name’ is reconstructible to Proto-Uralic, the word must have transferred already during the third millennium BC and consequently in the earliest stratum of borrowings. It is possible that the early Proto-Turkic speech community adopted the full compound of ‘name-fame’ in the same period where ‘seven’ and ‘sister-in-law’ was borrowed, although this remains to be verified, and a later transfer into Common Turkic from Tocharian remains a possibility.

### Sister-in-law

The widespread word for a ‘female in-law’ may similarly be hypothesised to have transferred into the Uralo-Turkic linguistic area (including Tungusic and Yukaghir) with the advent of an Indo-European speaking Afanasievo culture. A reasonable explanation for its popular status is an ingrained token of alliances facilitated between different clans in a ‘strictly exogamous, hierarchical, patrilocal and patrilineal system typical of pastoral societies being indigenous to the ancient Indo-European peoples’ (Olsen, 2020: 134–136, 162). It is noteworthy that this item is the only relational term not internally derived in Indo-European. The item must have been borrowed at the Proto-Uralic stage, suggesting that the word transferred in the third millennium BC, after the emergence of Afanasievo, but before the ultimate dissolution of Proto-Uralic, no later than the onset of the Seima–Turbino phenomenon ca. 2100 BC.

### Honey

This item is the most difficult to tie directly to demonstrable innovations in Central Asia. The obvious Indo-European provenance and significant spread among the Central and East Asian languages nonetheless point to a related phenomenon, perhaps with agriculture (Roffet-Salque et al., 2015; Bjørn, 2017, 95). While Uralic borrowed the word from an early Indo-European dialect (either early Indo-Iranic, as commonly thought, or the language of Afanasievo if older), the Proto-Turkic peoples adopted the word from an Old Iranic language. The late entry to Old Chinese either belongs to the Uralic tradition or represents a separate transfer. These different considerations make a clear relative chronology difficult to attain. If the presupposition that Proto-Samoyedic lost the word is accepted, the word must have transferred in the Afanasievo phase, but since there is no real evidence for this, a more solid *terminus ante quem* is the spread of Seima–Turbino ca. 2100 BC. Early Iranic contacts with Proto-Turkic are confined to a window between the incursion of Andronovo (early second millennium BC) and the split of the Turkic language (middle to late first millennium BC). The borrowing into Old Chinese is fairly securely dated to the last half of the first millennium BC, but the direct source language cannot be identified.

### Gold, copper, bronze

Designating a very tangible piece of the archaeological record, both Uralic and Turkic point to early adoption of the term from the Indo-European word for ‘gold’ that are continued both as ‘copper, brass’ and ‘iron, metal’. These shifts in meaning have to be considered in the context of recurring metallurgical innovations, naturally starting with the adoption of ‘a metal’ from Afanasievo, where both gold and bronze (as well as silver) have been found (Mallory & Adams, 1997: 4–6). Since neither Uralic nor Turkic continue the actual meaning ‘gold’, it is conceivable that the shift in meaning happened with the initial borrowing as the more common ‘bronze’ (Rédei, 1983, pp. 218-219). Okunevo, the successor culture of Afanasievo, notably improved upon the bronze technology, which is continued and spread with the Seima–Turbino phenomenon (Mei, 2003). However, the Uralic comparanda are not internally regular, and point to a spread after the formal dissolution of Proto-Uralic, but still early enough to have spread throughout the family, so likely after the adoption of both ‘seven’ and ‘name’. The irregular correspondences may alternatively be explained through re-borrowings from different centres, thus having ‘gold/metal/bronze’ transferred already with Afanasievo. The introduction of iron is a relatively late event that triggered a secondary shift in Proto-Samoyedic. The fact that the earliest Turkic tribes were believed to have been expert metallurgists in the Altai mountains (Golden, 2006: 141–142) only strengthens the spatial, temporal and cultural ties to Uralic and the introduction of metals through the Afanasievo communities.

### Horse (and chariot)

An Old Iranic source for the ‘horse’ in Old Chinese fits both the historical import of the most excellent horses from the Ferghana Valley into China and the archaeological observation that horse use dramatically expanded with the incursion of Andronovo beginning ca. 1900 BC (Jeong et al., 2020: 893–894; Shaughnessy, 1988). This later date, spreading from Sintashta and into Central and East Asia with Andronovo has recently been substantiated with genetic evidence (Librado et al., 2021), which also helps explain why horse terminology was not adopted into the eastern steppe zone, and linguistically into Uralic or Turkic, with the Afanasievo spread (Taylor, 2021; Honeychurch et al., 2021). The spread of Sintashta-derived domesticated horses perhaps supplanted earlier and less sophisticated horse usage, since also earlier branches of Indo-European attest to a common word and must all have been well acquainted with the horse continually since they broke away from Proto-Indo-European. The primary branches in Uralic later borrowed their terms independently, e.g. Proto-Samoyedic *juntз from Common Turkic *junt ‘horse, mare’ (if not both from a common substrate, Vovin, 2004: 126–127), while the Proto-Turkic form *(h)at appears unique. This situation also provides a valuable perspective of the intricate prehistoric contact situation between Pre-Proto-Turkic and Pre-Proto-Mongolic: since Turkic appears to have been indifferent to both Tocharian and Iranic horses, the Mongolic borrowing suggests that the two communities did not adopt horses at the same time or from the same source. With an Iranic source, a *terminus post quem* can be established with the spread of the Andronovo horizon, and even more succinct dating and location probably follow the earliest adoption of the horse and chariot in the Old Chinese speech community. The suggestion by Lubotsky (1998) of Tocharian origins of the chariot vocabulary in Old Chinese is consequently impeded by the same set of findings. The linguistic comparison is challenged by rather straightforward internal derivations in Old Chinese (Sagart, 1999: 204) and the evidence for a specialised industry in Afanasievo is wanting in comparison with the later diffusion of the horse and chariot tradition perfected in Sintashta and the subsequent Andronovo-driven spread of the complex across the steppes and into China (Librado et al., 2021; Shelach-Lavi, 2015; Rawson et al., 2020; Honeychurch et al., 2021; Shaughnessy, 1988).

### The origin of the Tocharian languages

The assumption that the Tocharian languages of the Tarim Basin ultimately derive from Afanasievo is in principle unnecessary for the formulation of the linguistic heritage of the Afanasievo culture. The connection is nonetheless suggested by linguistic phenomena shared with Uralic (see above), as well as a small, but suggestive set of potential Turkic borrowings (Lubotsky & Starostin, 2003). The most parsimonious solution to the emergence of the Tocharian languages in Central Asia is consequently from a (central or southern) dialect of the language of Afanasievo.

Treating the language of Afanasievo as distinct from its likely descendant Tocharian provides a framework for dealing with the earliest stages of Indo-European presence and contact in Central Asia, and can be extended to cover extinct branches surviving long enough to leave a mark as adstrate in extant languages, thus early loanwords in Uralic and Turkic not directly compatible with the Tocharian evidence. The language of Afanasievo suffered almost complete language extinction owing to both Iranic and Turkic encroachment, eclipsing most dialects before they had the chance to be documented; only Tocharian, by virtue of undertaking a second migration south into the Tarim Basin and the cultural sphere of Buddhist and Chinese written traditions, emerged before going extinct.

## Conclusion

The linguistic leg of the triangulation of human prehistory provides unique insights into the complex prehistory of Bronze Age Central Asia. The genetic and archaeological data are thus in agreement with the linguistic data to establish how the Afanasievo horizon brought the western Bronze Age to the Altai–Sayan region. The period from the introduction of Afansievo to the latest possible date of Proto-Uralic at the onset of the Seima–Turbino phenomenon constitutes a ‘Goldilocks zone’, neither too early for the development of Indo-European derived Bronze Age terminology, neither too late for borrowing at the Proto-Uralic stage. This period stretches from around 3300 to 2100 BC. Here, early stages of Uralic and Turkic engaged with the immigrants in trade and social reorganisation, prompting the adoption of the numeral ‘seven’ (that perhaps, albeit by unclear channels, was transmitted into Old Chinese) and eventually a particular concept of ‘name-fame’ and strategic intermarriage reflected in the term ‘sister-in-law’. The increased interaction had profound impact on the local communities as the adoption and specialisation in metallurgical techniques (borrowing ‘metal’) earned them a new role in the changing social structures on the steppe periphery: while parts of the Uralic grouping ventured west with the Seima–Turbino phenomenon and into even more lucrative exchange systems with the Indo-Iranic speech community, early speakers of Turkic eventually abandoned the trade to engage in the power struggles of the emerging pastoralist steppe confederations. With the incursion of the later Iranic speaking Andronovo culture did the use of horses markedly expand in the eastern steppe zone, prompting speakers of Old Chinese to borrow an Old Iranic word for ‘horse’.

As the loan hypotheses thus have been confirmed with each additional data point discovered, the linguistic testimony to the arrival of the Bronze Age in Central Asia provide crucial moorings for further exploration of the diverse language communities and cultural transformations of ancient Central Asia. The Indo-European identity of the Afanasievo culture finds linguistic substantiation, which adds further weight to the proposition that Tocharian languages derive from this early migration. Proto-Uralic was probably spoken in the Okunevo culture that constitutes the latest possible period for adoption of loanwords before the formation of the separate branches. The analysis also supports the proposition that an early stage of the Proto-Turkic language community was present around the Altai Mountains before 2000 BC, probably reflected in the eastern genetic component of Okunevo, and surely in the culturally transmitted loanwords of Indo-European provenance shared with early Uralic. Lastly, the identification of ‘seven’ as a Wanderwort potentially reaching early Proto-Sinitic may help date the precipitation of Old Chinese and contextualise the development of the Sino-Tibetan numeral system(s).

## Research transparency and reproducibility

All the relevant data is presented in the article. Further details are available in the referenced works.
